# *N*,*N*′-[Oxybis(benzene-4,1-di­yl)]diacetamide

**DOI:** 10.1107/S2414314625003840

**Published:** 2025-05-02

**Authors:** Rao M. Uppu, Ogad A. Agu, Sainath Babu, Patrick F. Mensah, Frank R. Fronczek

**Affiliations:** a Department of Environmental Toxicology Southern University and A&M College Baton Rouge Louisiana 70813 USA; b Department of Mechanical Engineering Southern University and A&M College Baton Rouge Louisiana 70813 USA; c Department of Chemistry Louisiana State University,Baton Rouge Louisiana 70803 USA; University of Aberdeen, United Kingdom

**Keywords:** crystal structure, acetamino­phen impurity N, anilide analgesics, *N*,*N*′-(oxybis(4,1-phenyl­ene))diacetamide

## Abstract

In the title compound, C_16_H_16_N_2_O_3_, the phenyl groups are twisted away from coplanarity with the ether linkage, forming a dihedral angle of 59.49 (4)° with each other. The ether oxygen atom lies somewhat out of both phenyl planes, by 0.066 (2) and 0.097 (2) Å. The acetamide substituents have quite different conformations with respect to the phenyl groups on either side of the mol­ecule. On one side, the C—C—N—C torsion angle is 21.0 (2)°, while on the other side it is 76.4 (2)°. In the crystal, the acetamide N—H groups form inter­molecular N—H⋯O hydrogen bonds to acetamide O atom, with both NH groups donating to the same mol­ecule. Thus, ladder-like chains exist in the [101] direction. One of the methyl groups has its H atoms disordered into two orientations, and the crystal chosen for data collection was found to be twinned.

## Abstract 2

In the title compound, the phenyl groups are twisted away from coplanarity with the ether linkage, forming a dihedral angle of 59.49 (4)° with each other. In the crystal, the acetamide N—H groups form inter­molecular N—H⋯O hydrogen bonds to acetamide O atom, with both NH groups donating to the same mol­ecule. Thus, ladder-like chains exist in the [101] direction.

## Graphical abstract

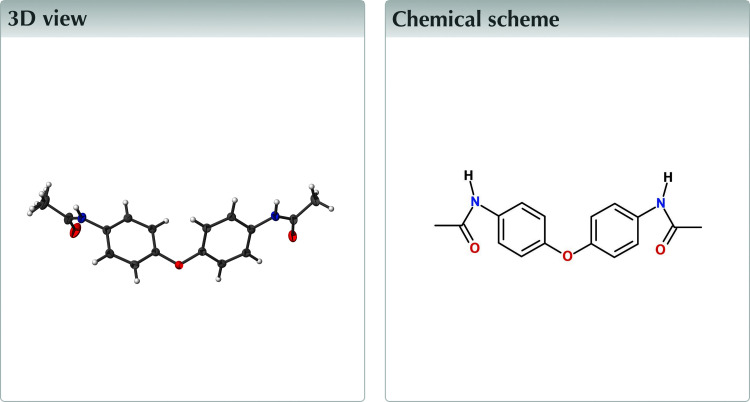

## Structure description

Acetamino­phen (*N*-[4-hy­droxy­phen­yl]acetamide, C_8_H_9_NO_2_), also known by various brand names such as Tylenol or Panadol in different countries, ranks among the most widely used pain relievers and fever reducers worldwide (Bertolini *et al.*, 2006 ▸; Ohashi & Kohno, 2020 ▸). Introduced in the 1950s, current estimates show that over 25 billion doses are sold each year in the United States alone (Yoon *et al.*, 2016 ▸). While this underscores the importance of acetamino­phen in over-the-counter pain management, as with most medications, the focus extends beyond the safety and efficacy of the active pharmaceutical ingredient (API). Regulatory agencies also pay close attention to impurities, particularly those present in small amounts but still capable of raising concerns (ICH, 2006*a* ▸,*b* ▸). Although these impurities are generally not expected to cause immediate harm, about 50,000 emergency room visits in the United States each year are linked to acetamino­phen toxicity, which can result in severe liver damage (specifically, centrilobular necrosis) and, in some cases, death (Stravitz & Lee, 2019 ▸; Yoon *et al.*, 2016 ▸). In recent years, the emphasis on monitoring even trace levels of impurities has increased, given their potential impact on both effectiveness of the drug and its long-term safety (ICH, 2006*a* ▸, 2021 ▸).

The title compound, *N*,*N*′-(oxydi­benzene-4,1-di­yl)diacetamide (C_16_H_16_N_2_O_3_), commonly known as Impurity N, is one of over a dozen potential byproducts that can occur in acetamino­phen. Typically present at levels below 0.1% of the active pharmaceutical ingredient (API) (Arıkan *et al.*, 2023 ▸), Impurity N forms when 4-amino­phenol undergoes oxidative coupling during the manufacturing process. In standard industrial practice, 4-amino­phenol is acetyl­ated to produce acetamino­phen; however, if two 4-amino­phenol mol­ecules couple oxidatively, they form 4,4′-oxydianiline, which then becomes Impurity N upon acetyl­ation (NCBI, 2025 ▸). Even minimal traces of 4-amino­phenol that dimerize or incomplete reduction of 4-nitro­phenol can introduce this impurity. Additionally, under certain oxidizing conditions during storage, two acetamino­phen mol­ecules can theoretically couple *via* their phenolic –OH groups, creating the same ether-linked dimer (Rao & Narasaraju, 2006 ▸). These pathways are generally minor, and robust process controls combined with proper storage conditions typically keep Impurity N at trace levels (Kamberi *et al.*, 2004 ▸). Various analytical methods, such as reversed-phase HPLC or UPLC coupled with UV–Vis spectroscopy, photodiode array, or mass spectrometry detection are used to detect Impurity N with sensitivity down to p.p.m. or sub-p.p.m., ensuring that its presence remains within acceptable limits in the final acetamino­phen batches (Arıkan *et al.*, 2023 ▸).

Impurity N currently lacks any toxicological or pharmacological characterization. However, by analogy to the metabolism of acetamino­phen and other 4-alk­oxy­anilides, this impurity is likely to undergo partial or complete de­acetyl­ation (Nohmi *et al.*, 1984 ▸; Ohashi & Kohno, 2020 ▸; Prescott, 1980 ▸). Such metabolism would yield aromatic amine derivatives, notably *N*[4-(4-amino­phen­oxy)phen­yl]acetamide (the mono-de­acetyl­ated product) and 4,4′-oxydianiline (the fully de­acetyl­ated di­amine). In turn, these aromatic amines could undergo further biotransformations analogous to those of 4-amino­phenol and 4-alk­oxy­aniline, potentially forming *N*-arachidonoylphenolamine (AM404)-like anandamide analogues or 4-alk­oxy­nitro­sophenol derivatives (Ohashi & Kohno, 2020 ▸; Zygmunt *et al.*, 2000 ▸). Metabolites of this type are known to elicit diverse pharmacological and pathophysiological effects. For example, certain 4-alk­oxy­aniline metabolites can inhibit cyclo­oxygenase-1 (COX-1) and have demonstrated carcinogenic and nephrotoxic effects (Kankuri *et al.*, 2003 ▸; NTP, 1990 ▸; Togei *et al.*, 1987 ▸). These metabolic considerations suggest that Impurity N could similarly give rise to bioactive or toxic species, warranting further toxicological evaluation, despite the current lack of direct data. To better understand the mol­ecular structure and to inform studies of its potential biological inter­actions, we crystallized Impurity N from aqueous solution and determined its structure *via* single-crystal X-ray diffraction.

**Figure 1:**
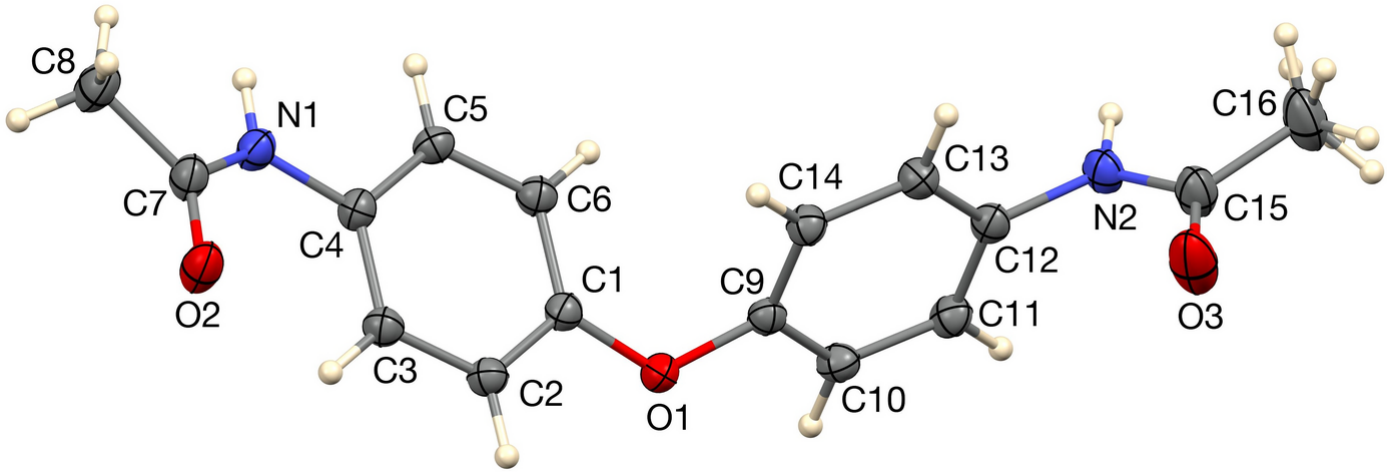
The asymmetric unit of the title compound with 50% ellipsoids.

The title compound, C_16_H_16_N_2_O_3_ crystallizes with one mol­ecule in the asymmetric unit (Fig. 1 ▸) in space group *P*2_1_/*n*. The C1–C6 and C9–C14 phenyl groups are twisted out of coplanarity with the ether linkage, forming a dihedral angle of 59.49 (4)° with each other. The ether oxygen atom, O1, lies slightly out of the planes of both phenyl rings, by 0.066 (2) and 0.097 (2) Å, respectively. The acetamide substituents adopt markedly different conformations relative to the adjacent phenyl groups. On one side of the mol­ecule, the C3—C4—N1—C7 torsion angle is 21.0 (2)°, while on the opposite side, the C13—C12—N2—C15 angle is 76.4 (2)°.

**Figure 2:**
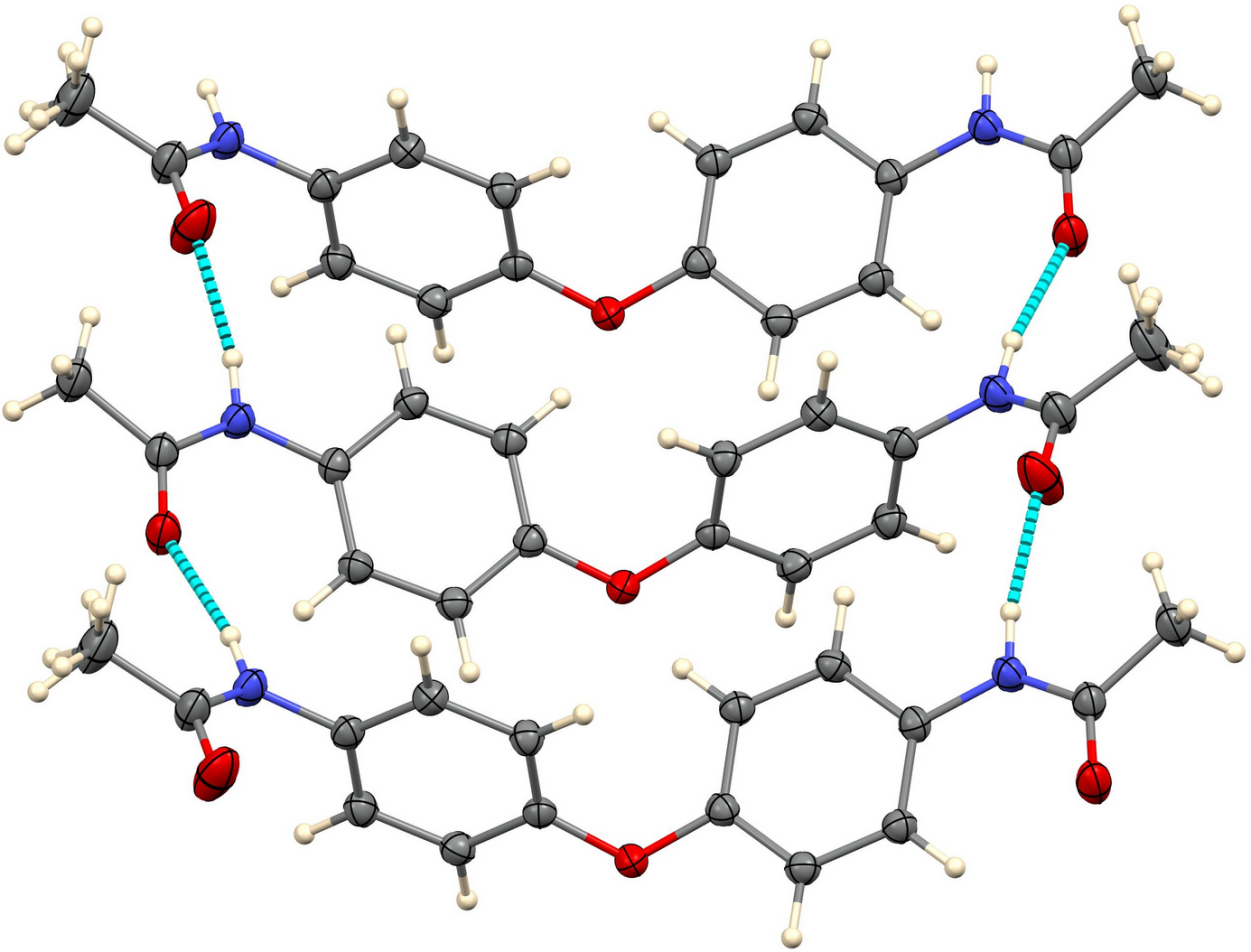
Detail of the the hydrogen bonding with 50% ellipsoids.

**Figure 3:**
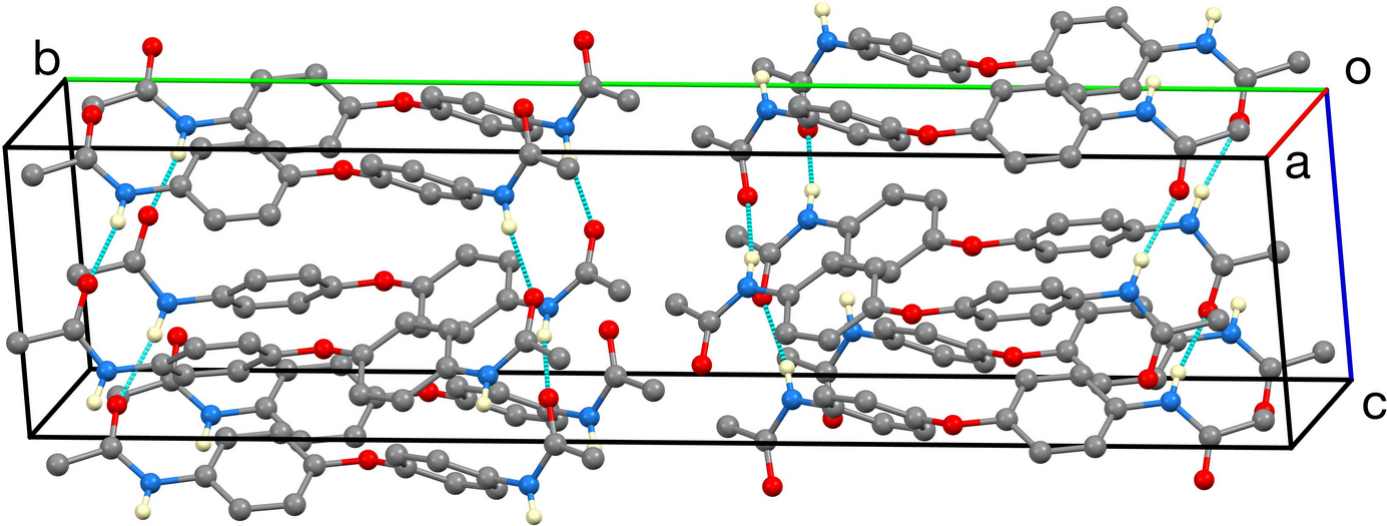
The unit-cell packing. Only NH hydrogen atoms are shown.

In the extended structure, the acetamide N—H groups participate in N—H⋯O hydrogen bonds (Table 1 ▸), each donating to the carbonyl oxygen atom of another acetamide group. Both N—H donors inter­act with the same acceptor mol­ecule at *x* + 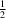, 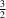 − *y*, 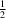 + *z*, resulting in the formation of ladder-like chains extending along the [101] direction, as shown in Fig. 2 ▸. The N⋯O distances in these hydrogen bonds are 2.834 (2) and 2.9066 (18) Å. One of the methyl groups exhibits hydrogen-atom disorder over two orientations, and the crystal was a pseudomerohedral twin. The unit-cell packing is illustrated in Fig. 3 ▸.

## Synthesis and crystallization

*N*,*N*′-(Oxydi­benzene-4,1-di­yl)diacetamide, C_16_H_16_N_2_O_3_ (CAS 3070–86-8) was obtained from AmBeed (Arlington Heights, Illinois, USA) and was used without further purification. Crystals in the form of colorless laths were prepared by slow cooling of a nearly saturated solution of the title compound in boiling deionized water (resistance *ca*. 18 *M*Ω cm^−1^).

## Refinement

Crystal data, data collection and structure refinement details are summarized in Table 2 ▸. The crystal was a slight pseudomerohedral twin with twin law [1 0 0, 0 −1 0, 0 0 −1] and refined BASF parameter of 0.0053 (5). All H atoms were located in difference maps and those on C were thereafter treated as riding in geometrically idealized positions with C—H distances 0.95 Å for phenyl and 0.98 Å for methyl. Coordinates of the N—H atom were refined. *U*_iso_(H) values were assigned as 1.2*U*_eq_ for the attached atom (1.5 for meth­yl). The H atoms on methyl group C16 were disordered into two conformations and were treated as two half-occupied sets related by a 60° torsional rotation. A residual density peak of 0.90 e Å^−3^ lies 0.95 Å from the O atom (O3) of the acetamide containing the disordered methyl group, perhaps indicative of further disorder in this substituent or imperfect handling of the twinning. The 0 4 0 reflection was omitted from the refinement, having negative *F*_o_ and large *F*_c_.

## Supplementary Material

Crystal structure: contains datablock(s) I. DOI: 10.1107/S2414314625003840/hb4515sup1.cif

Structure factors: contains datablock(s) I. DOI: 10.1107/S2414314625003840/hb4515Isup2.hkl

Supporting information file. DOI: 10.1107/S2414314625003840/hb4515Isup3.cml

CCDC reference: 2447482

Additional supporting information:  crystallographic information; 3D view; checkCIF report

## Figures and Tables

**Table 1:** Hydrogen-bond geometry (Å, °)

*D*—H⋯*A*	*D*—H	H⋯*A*	*D*⋯*A*	*D*—H⋯*A*
N1—H1*N*⋯O3^i^	0.88 (2)	1.96 (2)	2.834 (2)	169.5 (19)
N2—H2*N*⋯O2^i^	0.88 (2)	2.03 (2)	2.9066 (18)	170 (2)

Symmetry code: (i) 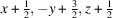.

**Table 2:** Experimental details

Crystal data	
Chemical formula	C_16_H_16_N_2_O_3_
*M*_r_	284.31
Crystal system, space group	Monoclinic, *P*2_1_/*n*
Temperature (K)	100
*a*, *b*, *c* (Å)	5.5676 (5), 33.185 (3), 7.6949 (5)
β (°)	90.325 (2)
*V* (Å^3^)	1421.68 (19)
*Z*	4
Radiation type	Ag *K*α, λ = 0.56086 Å
μ (mm^−1^)	0.06
Crystal size (mm)	0.32 × 0.27 × 0.09

Data collection	
Diffractometer	Bruker D8 Venture DUO with Photon III C14
Absorption correction	Multi-scan (*SADABS*; Krause *et al.*, 2015 ▸)
*T*_min_, *T*_max_	0.933, 0.995
No. of measured, independent and observed [*I* > 2σ(*I*)] reflections	22399, 4306, 3468
*R*_int_	0.065
(sin θ/λ)_max_ (Å^−1^)	0.714

Refinement	
*R*[*F*^2^ > 2σ(*F*^2^)], *wR*(*F*^2^), *S*	0.065, 0.179, 1.05
No. of reflections	4306
No. of parameters	198
H-atom treatment	H atoms treated by a mixture of independent and constrained refinement
Δρ_max_, Δρ_min_ (e Å^−3^)	0.90, −0.43

Computer programs: *APEX5* and *SAINT* (Bruker, 2016 ▸), *SHELXT2018/2* (Sheldrick, 2015 ▸a), *SHELXL2019/1* (Sheldrick, 2015 ▸b), *Mercury* (Macrae *et al.*, 2020 ▸) and *publCIF* (Westrip, 2010 ▸).

## full crystallographic data

### N,N'-[Oxybis(benzene-4,1-diyl)]diacetamide Crystal data

**Table:**

C_16_H_16_N_2_O_3_	*F*(000) = 600
*M**_r_* = 284.31	*D*_x_ = 1.328 Mg m^−^^3^
Monoclinic, *P*2_1_/*n*	Ag *K*α radiation, λ = 0.56086 Å
*a* = 5.5676 (5) Å	Cell parameters from 4722 reflections
*b* = 33.185 (3) Å	θ = 2.5–23.6°
*c* = 7.6949 (5) Å	µ = 0.06 mm^−^^1^
β = 90.325 (2)°	*T* = 100 K
*V* = 1421.68 (19) Å^3^	Plate, colourless
*Z* = 4	0.32 × 0.27 × 0.09 mm

### N,N'-[Oxybis(benzene-4,1-diyl)]diacetamide Data collection

**Table:**

Bruker D8 Venture DUO with Photon III C14 diffractometer	3468 reflections with *I* > 2σ(*I*)
Radiation source: IµS 3.0 microfocus	*R*_int_ = 0.065
φ and ω scans	θ_max_ = 23.6°, θ_min_ = 2.1°
Absorption correction: multi-scan (SADABS; Krause *et al.*, 2015)	*h* = −7→5
*T*_min_ = 0.933, *T*_max_ = 0.995	*k* = −47→47
22399 measured reflections	*l* = −10→10
4306 independent reflections	

### N,N'-[Oxybis(benzene-4,1-diyl)]diacetamide Refinement

**Table:**

Refinement on *F*^2^	0 restraints
Least-squares matrix: full	Hydrogen site location: mixed
*R*[*F*^2^ > 2σ(*F*^2^)] = 0.065	H atoms treated by a mixture of independent and constrained refinement
*wR*(*F*^2^) = 0.179	*w* = 1/[σ^2^(*F*_o_^2^) + (0.0799*P*)^2^ + 0.725*P*] where *P* = (*F*_o_^2^ + 2*F*_c_^2^)/3
*S* = 1.05	(Δ/σ)_max_ = 0.001
4306 reflections	Δρ_max_ = 0.90 e Å^−^^3^
198 parameters	Δρ_min_ = −0.43 e Å^−^^3^

### N,N'-[Oxybis(benzene-4,1-diyl)]diacetamide Special details

**Table:**

Geometry. All esds (except the esd in the dihedral angle between two l.s. planes) are estimated using the full covariance matrix. The cell esds are taken into account individually in the estimation of esds in distances, angles and torsion angles; correlations between esds in cell parameters are only used when they are defined by crystal symmetry. An approximate (isotropic) treatment of cell esds is used for estimating esds involving l.s. planes.
Refinement. Refined as a 2-component twin.

### N,N'-[Oxybis(benzene-4,1-diyl)]diacetamide Fractional atomic coordinates and isotropic or equivalent isotropic displacement parameters (Å^2^)

**Table:**

	*x*	*y*	*z*	*U*_iso_*/*U*_eq_	Occ. (<1)
O1	0.1938 (2)	0.75069 (3)	0.64144 (15)	0.0233 (2)	
O2	0.4346 (2)	0.56780 (3)	0.40229 (16)	0.0333 (3)	
O3	0.5307 (3)	0.91576 (5)	0.3437 (2)	0.0510 (4)	
N1	0.6286 (3)	0.59932 (4)	0.62577 (17)	0.0247 (3)	
H1N	0.756 (4)	0.5980 (6)	0.695 (3)	0.030*	
N2	0.6478 (3)	0.90044 (4)	0.61679 (18)	0.0263 (3)	
H2N	0.744 (4)	0.9074 (6)	0.703 (3)	0.032*	
C1	0.3149 (3)	0.71413 (4)	0.63352 (18)	0.0209 (3)	
C2	0.1942 (3)	0.68320 (4)	0.54986 (19)	0.0222 (3)	
H2A	0.045052	0.688389	0.493585	0.027*	
C3	0.2906 (3)	0.64448 (4)	0.54791 (19)	0.0228 (3)	
H3	0.205855	0.623136	0.492738	0.027*	
C4	0.5119 (3)	0.63719 (4)	0.62715 (19)	0.0215 (3)	
C5	0.6302 (3)	0.66855 (4)	0.71332 (19)	0.0218 (3)	
H5	0.779782	0.663529	0.769297	0.026*	
C6	0.5316 (3)	0.70702 (4)	0.71806 (19)	0.0223 (3)	
H6	0.611423	0.728157	0.778366	0.027*	
C7	0.5930 (3)	0.56846 (4)	0.5137 (2)	0.0261 (3)	
C8	0.7694 (4)	0.53426 (5)	0.5312 (3)	0.0348 (4)	
H8A	0.893903	0.541497	0.616144	0.052*	
H8B	0.843772	0.529005	0.418365	0.052*	
H8C	0.685113	0.510015	0.570580	0.052*	
C9	0.3190 (3)	0.78663 (4)	0.63494 (18)	0.0204 (3)	
C10	0.2098 (3)	0.81915 (5)	0.7162 (2)	0.0234 (3)	
H10	0.063804	0.815553	0.777608	0.028*	
C11	0.3159 (3)	0.85704 (4)	0.7068 (2)	0.0246 (3)	
H11	0.240517	0.879529	0.759981	0.030*	
C12	0.5310 (3)	0.86207 (4)	0.62024 (19)	0.0228 (3)	
C13	0.6378 (3)	0.82935 (4)	0.53787 (19)	0.0230 (3)	
H13	0.784291	0.832953	0.477080	0.028*	
C14	0.5318 (3)	0.79144 (4)	0.54388 (19)	0.0222 (3)	
H14	0.603747	0.769176	0.486610	0.027*	
C15	0.6455 (4)	0.92400 (5)	0.4754 (2)	0.0335 (4)	
C16	0.8005 (4)	0.96122 (6)	0.4857 (3)	0.0458 (5)	
H16A	0.877402	0.962618	0.600446	0.069*	0.5
H16B	0.924151	0.960014	0.395741	0.069*	0.5
H16C	0.700668	0.985194	0.467726	0.069*	0.5
H16D	0.790745	0.975933	0.375496	0.069*	0.5
H16E	0.743996	0.978537	0.580201	0.069*	0.5
H16F	0.967479	0.953357	0.508215	0.069*	0.5

### N,N'-[Oxybis(benzene-4,1-diyl)]diacetamide Atomic displacement parameters (Å^2^)

**Table:**

	*U*^11^	*U*^22^	*U*^33^	*U*^12^	*U*^13^	*U*^23^
O1	0.0205 (5)	0.0192 (5)	0.0303 (6)	0.0009 (4)	−0.0001 (4)	0.0008 (4)
O2	0.0427 (7)	0.0221 (5)	0.0349 (6)	0.0029 (5)	−0.0140 (5)	−0.0010 (4)
O3	0.0667 (11)	0.0395 (8)	0.0464 (8)	−0.0211 (7)	−0.0289 (8)	0.0121 (6)
N1	0.0271 (7)	0.0211 (6)	0.0257 (6)	0.0031 (5)	−0.0055 (5)	0.0009 (5)
N2	0.0302 (7)	0.0218 (6)	0.0269 (7)	−0.0051 (5)	−0.0066 (5)	−0.0020 (5)
C1	0.0207 (7)	0.0202 (6)	0.0219 (6)	0.0009 (5)	0.0003 (5)	0.0019 (5)
C2	0.0203 (7)	0.0236 (7)	0.0228 (7)	−0.0002 (5)	−0.0016 (5)	0.0006 (5)
C3	0.0225 (7)	0.0225 (7)	0.0235 (7)	−0.0012 (5)	−0.0025 (5)	0.0003 (5)
C4	0.0235 (7)	0.0198 (6)	0.0212 (7)	0.0013 (5)	−0.0003 (5)	0.0014 (5)
C5	0.0209 (7)	0.0231 (7)	0.0215 (6)	0.0012 (5)	−0.0023 (5)	0.0011 (5)
C6	0.0227 (7)	0.0221 (7)	0.0219 (7)	−0.0002 (5)	−0.0017 (5)	−0.0003 (5)
C7	0.0313 (8)	0.0194 (6)	0.0275 (7)	0.0011 (6)	−0.0039 (6)	0.0013 (5)
C8	0.0406 (10)	0.0238 (7)	0.0399 (9)	0.0080 (7)	−0.0109 (8)	−0.0036 (6)
C9	0.0201 (6)	0.0201 (6)	0.0210 (6)	0.0003 (5)	−0.0035 (5)	−0.0001 (5)
C10	0.0211 (7)	0.0244 (7)	0.0247 (7)	0.0022 (5)	−0.0006 (5)	−0.0021 (5)
C11	0.0256 (7)	0.0221 (7)	0.0261 (7)	0.0029 (5)	−0.0020 (6)	−0.0043 (5)
C12	0.0249 (7)	0.0201 (6)	0.0231 (7)	−0.0007 (5)	−0.0052 (5)	−0.0014 (5)
C13	0.0226 (7)	0.0226 (7)	0.0237 (7)	−0.0005 (5)	0.0001 (5)	0.0000 (5)
C14	0.0230 (7)	0.0213 (6)	0.0224 (7)	0.0009 (5)	0.0013 (5)	−0.0010 (5)
C15	0.0399 (10)	0.0250 (8)	0.0355 (9)	−0.0077 (7)	−0.0111 (7)	0.0029 (6)
C16	0.0603 (13)	0.0322 (9)	0.0446 (11)	−0.0202 (9)	−0.0143 (10)	0.0058 (8)

### N,N'-[Oxybis(benzene-4,1-diyl)]diacetamide Geometric parameters (Å, º)

**Table:**

O1—C9	1.3827 (17)	C8—H8A	0.9800
O1—C1	1.3896 (17)	C8—H8B	0.9800
O2—C7	1.227 (2)	C8—H8C	0.9800
O3—C15	1.226 (2)	C9—C10	1.389 (2)
N1—C7	1.353 (2)	C9—C14	1.389 (2)
N1—C4	1.4150 (19)	C10—C11	1.392 (2)
N1—H1N	0.88 (2)	C10—H10	0.9500
N2—C15	1.340 (2)	C11—C12	1.384 (2)
N2—C12	1.4299 (19)	C11—H11	0.9500
N2—H2N	0.88 (2)	C12—C13	1.392 (2)
C1—C2	1.384 (2)	C13—C14	1.391 (2)
C1—C6	1.388 (2)	C13—H13	0.9500
C2—C3	1.393 (2)	C14—H14	0.9500
C2—H2A	0.9500	C15—C16	1.508 (2)
C3—C4	1.392 (2)	C16—H16A	0.9800
C3—H3	0.9500	C16—H16B	0.9800
C4—C5	1.397 (2)	C16—H16C	0.9800
C5—C6	1.390 (2)	C16—H16D	0.9800
C5—H5	0.9500	C16—H16E	0.9800
C6—H6	0.9500	C16—H16F	0.9800
C7—C8	1.506 (2)		
			
C9—O1—C1	120.45 (12)	H8A—C8—H8C	109.5
C7—N1—C4	127.70 (13)	H8B—C8—H8C	109.5
C7—N1—H1N	117.3 (14)	O1—C9—C10	115.59 (13)
C4—N1—H1N	114.1 (13)	O1—C9—C14	123.29 (13)
C15—N2—C12	122.17 (14)	C10—C9—C14	120.97 (13)
C15—N2—H2N	117.5 (14)	C9—C10—C11	119.45 (14)
C12—N2—H2N	119.5 (14)	C9—C10—H10	120.3
C2—C1—C6	120.72 (13)	C11—C10—H10	120.3
C2—C1—O1	115.69 (13)	C12—C11—C10	120.20 (14)
C6—C1—O1	123.27 (13)	C12—C11—H11	119.9
C1—C2—C3	120.20 (14)	C10—C11—H11	119.9
C1—C2—H2A	119.9	C11—C12—C13	119.87 (14)
C3—C2—H2A	119.9	C11—C12—N2	120.73 (13)
C4—C3—C2	119.72 (14)	C13—C12—N2	119.39 (14)
C4—C3—H3	120.1	C14—C13—C12	120.53 (14)
C2—C3—H3	120.1	C14—C13—H13	119.7
C3—C4—C5	119.48 (13)	C12—C13—H13	119.7
C3—C4—N1	123.81 (13)	C9—C14—C13	118.95 (13)
C5—C4—N1	116.72 (13)	C9—C14—H14	120.5
C6—C5—C4	120.73 (14)	C13—C14—H14	120.5
C6—C5—H5	119.6	O3—C15—N2	122.93 (16)
C4—C5—H5	119.6	O3—C15—C16	121.45 (16)
C1—C6—C5	119.09 (13)	N2—C15—C16	115.60 (16)
C1—C6—H6	120.5	C15—C16—H16A	109.5
C5—C6—H6	120.5	C15—C16—H16B	109.5
O2—C7—N1	124.15 (14)	H16A—C16—H16B	109.5
O2—C7—C8	120.98 (14)	C15—C16—H16C	109.5
N1—C7—C8	114.85 (14)	H16A—C16—H16C	109.5
C7—C8—H8A	109.5	H16B—C16—H16C	109.5
C7—C8—H8B	109.5	H16D—C16—H16E	109.5
H8A—C8—H8B	109.5	H16D—C16—H16F	109.5
C7—C8—H8C	109.5	H16E—C16—H16F	109.5
			
C9—O1—C1—C2	−146.40 (13)	C1—O1—C9—C10	−153.03 (13)
C9—O1—C1—C6	40.1 (2)	C1—O1—C9—C14	31.3 (2)
C6—C1—C2—C3	−0.7 (2)	O1—C9—C10—C11	−176.08 (13)
O1—C1—C2—C3	−174.44 (13)	C14—C9—C10—C11	−0.3 (2)
C1—C2—C3—C4	−1.4 (2)	C9—C10—C11—C12	−1.3 (2)
C2—C3—C4—C5	2.4 (2)	C10—C11—C12—C13	1.9 (2)
C2—C3—C4—N1	−177.33 (14)	C10—C11—C12—N2	−176.98 (14)
C7—N1—C4—C3	21.0 (2)	C15—N2—C12—C11	−104.7 (2)
C7—N1—C4—C5	−158.72 (16)	C15—N2—C12—C13	76.4 (2)
C3—C4—C5—C6	−1.1 (2)	C11—C12—C13—C14	−0.9 (2)
N1—C4—C5—C6	178.57 (13)	N2—C12—C13—C14	178.00 (13)
C2—C1—C6—C5	1.9 (2)	O1—C9—C14—C13	176.73 (13)
O1—C1—C6—C5	175.17 (13)	C10—C9—C14—C13	1.3 (2)
C4—C5—C6—C1	−1.0 (2)	C12—C13—C14—C9	−0.7 (2)
C4—N1—C7—O2	−6.5 (3)	C12—N2—C15—O3	5.1 (3)
C4—N1—C7—C8	171.91 (15)	C12—N2—C15—C16	−173.16 (17)

### N,N'-[Oxybis(benzene-4,1-diyl)]diacetamide Hydrogen-bond geometry (Å, º)

**Table:**

*D*—H···*A*	*D*—H	H···*A*	*D*···*A*	*D*—H···*A*
N1—H1*N*···O3^i^	0.88 (2)	1.96 (2)	2.834 (2)	169.5 (19)
N2—H2*N*···O2^i^	0.88 (2)	2.03 (2)	2.9066 (18)	170 (2)

Symmetry code: (i) *x*+1/2, −*y*+3/2, *z*+1/2.
